# High levels of pyrethroid resistance and *super-kdr* mutations in the populations of tropical bed bug, *Cimex hemipterus*, in Iran

**DOI:** 10.1186/s13071-021-04962-5

**Published:** 2021-09-14

**Authors:** Mohammad Bagher Ghavami, Zarafat Ghahremani, Narges Raeisi, Behrooz Taghiloo

**Affiliations:** Department of Medical Entomology and Vector Control, School of Medicine, Zanjan University of Medical Sciences, Zanjan, Iran

**Keywords:** *Cimex hemipterus*, Tropical bed bug, *Kdr*/*super-kdr-*type mutations, Resistant to pyrethroids, Bioassay test

## Abstract

**Background:**

The tropical bed bug, *Cimex hemipterus*, is an important ectoparasite causing various health problems. This species is mainly confined to tropical regions; however, insecticide resistance, global warming, and globalization have changed its distribution map. Molecular information on pyrethroid resistance, which is essential for the development of control programs, is unknown for *C. hemipterus* in expanded areas. The present study was designed to determine the permethrin resistance status, characterize the pyrethroid receptor sites in voltage-gated sodium channel (*vgsc*) gene, and identify the resistance-related mutations in the populations of tropical bed bug in Iran.

**Methods:**

Live bed bugs were collected, and adults of *C. hemipterus* were selected for bioassay and molecular surveys. Bioassay was performed by tarsal contact with permethrin 0.75% in mixed-sex of samples. Conventional and quantitative TaqMan and SYBR Green real-time PCR assays were conducted to characterize the *vgsc* gene and genotypes of studied populations.

**Results:**

In the bioassay tests, the mortality rates were in the range of 30.7–38.7% and 56.2–77.4% in 24 and 48 h, respectively. The knockdown rates of studied populations were in the range of 32.2–46.6% and 61.5–83.8% in the first and second days, respectively. The KT_50_ and KT_90_ values in the *Cimex lectularius* Kh1 strain were presented as 5.39 and 15.55 h, respectively. These values in the selected populations of *C. hemipterus* varied from 27.9 to 29.5 and from 82.8 to 104.4 h, respectively. Knockdown time ratios (KR_50_ and KR_90_) in these populations varied from 5.17 to 6.17-fold compared with those of the *C. lectularius* Kh1 strain. Fragments of *vgsc* gene with 355 bp and 812 bp were amplified. Analysis of sequences revealed the A468T substitution, *kdr*-associated D953G, and *super-kdr* M918I and L1014F mutations in all populations.

**Conclusions:**

The specific/sensitive, safe, and rapid diagnostic assays developed in this study are recommended for detection of *kdr/super*-*kdr* mutations and frequency of mutant alleles. The presence of *super-kdr* mutations and high resistance to permethrin in all the populations necessitate the reconsideration of control approaches against *C. hemipterus*.

**Graphical Abstract:**

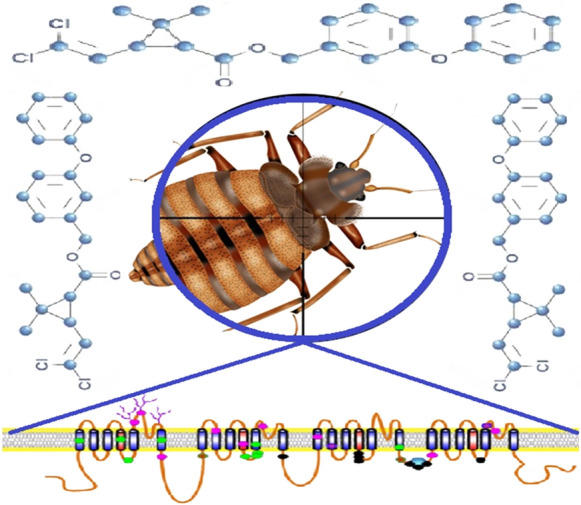

## Background

Bed bugs are important ectoparasites that commonly affect humans and are poised to become a major household pest throughout the world. Repeated bites by these hematophagous insects cause allergic reactions, including itching and erythematous or popular urticaria-like dermatitis, which favors secondary bacterial infections, cellulitis, impetigo, ecthyma, and lymphangitis. Infestation of these pests also gives rise to anxiety, insomnia, and worsening of an existing mental health condition. Bed bugs have been found to be naturally infected with more than 28 human pathogens; however, there is no substantial evidence of their pathogen transmission to humans through bites [1, 2]. Although hepatitis C virus (HCV) was recently identified in the common bed bug, the role of this bug as a potential vector for the transmission of HCV remains unclear [3].

Bed bugs have a long association with humans, and their infestation has dramatically been increased, especially during the past decades. Two cryptic species of bed bugs, the common bed bug, *Cimex lectularius*, and the tropical bed bug, *Cimex hemipterus*, are adapted to feed on humans, attack nocturnally, and affect all socioeconomic classes. The common bed bug is found in temperate climates, whereas the tropical bed bug is mainly confined to tropical regions. Nonetheless, global warming, globalization, international trade, and insecticide resistance are several factors that have changed the tropical bed bug distribution and triggered its emergence in subtropical and even temperate regions [4, 5]. Therefore, the Middle East and north of Africa are at high risk for infestation by this species. The tropical bed bug infestation has recently been reported from Israel and Kuwait [6, 7]. Spread of the tropical bed bug in these areas raises health issues, and besides, new species may be generated by the hybridization of its strains with the populations of the common bed bug.

Although non-chemical measures, such as environmental manipulation (cleaning and organizing the living areas, furniture, and house belongings and elimination of the bed bug hiding spots and clutter) and physical control (vacuuming, using barriers, such as mattresses, and applying thermal heat or cold treatments), could be helpful in reducing the number of bed bugs, their control has mainly relied on the application of insecticides [1–3]. In this context, pyrethroids have extensively been used for the control of bed bugs owing to their low mammalian toxicity, fast knockdown activity, and relatively low cost. However, the intensive and continuous application of these pesticides leads to resistance in bed bugs, posing a serious obstacle to their sustained effective control [8].

Earlier studies have shown that some insecticide-resistant bed bugs have multiple resistance mechanisms, including insensitivity of target sites*,* enhancement of metabolic detoxification enzymes, and reduction in the cuticular penetration [9]. An important resistance mechanism against pyrethroids and DDT is known as knockdown resistance (*kdr*). It has been evidenced that various mutations in the voltage-gated sodium channel (*vgsc*) gene are responsible for the increased resistance in many insect pests. These mutations change the amino acid sequences of the VGSC protein and decrease the insecticide effect on the nervous system [10–15].

Molecular analyses of susceptible and resistant strains of bed bugs have revealed that there are some point mutations in the *vgsc* gene and suggested that the substitution of the amino acids is the main reason for enhancing resistance to pyrethroids [12, 16–20]. Such alterations in the nervous cell membrane mitigate the effect of insecticide on the nervous system and cause resistance to paralysis. This intrinsic trait of *kdr*, which is associated with DDT and pyrethroids, was first identified in the housefly *Musca domestica* [21] and has been documented in different species of agricultural and public health pests [9–12, 20].

In bed bugs, *kdr*-type resistance to pyrethroids is associated with mutations in two VGSC regions (dual pyrethroid-receptor sites). The first region includes four putative *kdr* mutation sites, namely V410, V419, V421, and E435, encoding the domain IS6 and a part of the domain I–II linker [9, 19, 22–24]. The second region contains six putative *kdr* mutation sites, especially M918, L925, T929, L932, L936, and L1014 residues, encoding the domain IIS4-S6 region [9, 25–27], of which two M918I and L1014F mutations cause high resistance to pyrethroids in tropical bed bug. In this species, the M918I mutation is always found together with the L1014F mutation and probably plays a synergistic role in enhancing pyrethroid resistance [10, 13, 19]. Previous investigations have identified some novel mutations, such as L899V and D953G, in the *vgsc* gene of *C. hemipterus* [25] and six sequential (Y/L995H, A1007S, V1010L, I1011F, V1016E, and L1017F/S) mutations around the L1014F mutation [26]. However, the function of these mutations in pyrethroid resistance is unclear.

To date, numerous techniques have been reported for detecting *kdr* resistance in bed bug populations, but toxicity bioassay and molecular methods are conventional approaches in monitoring and expressing this resistance [9]. Bioassay methods are often unfeasible in routine monitoring of bed bugs as they need a sufficient number of live specimens, and obtaining a large number is difficult. Moreover, monitoring by these methods can be compromised, while the resistance alleles are recessive. Molecular techniques could provide an early warning of the development of insecticide resistance and could determine the specific resistance mechanism. Even though molecular assays such as PCR and direct DNA sequencing have been employed to detect *kdr* mutations in bed bug samples [23–25, 28], these methods are impracticable for studying large-scale populations. Accordingly, the development of conventional and sensitive quantitative real-time PCR (qRT-PCR) assays is necessary for detecting *kdr* mutations in field-collected strains.

In different areas of the Middle East, especially in Iran, along with other parts of the world, DDT and other organochlorine compounds (OCs; e.g. *gamma* HCH, dieldrin) have been exploited to control bed bugs since the 1950s. Following the OCs application, resistance to DDT has been reported from Iran, Israel, and Turkey in common bed bug [29–31]. Due to resistance development and environmental concern, the use of OCs was restricted in 1960s, and the progressive restriction and prohibition of such insecticides led to an increase in the use of organophosphates (OPs; e.g. malathion, diazinon, pirimiphos-methyl) and carbamates (Cs; e.g. carbaryl, propoxur, and bendiocarb) in the control plan for three decades. In Iran, pyrethroid insecticides (PYs; e.g. permethrin, cypermethrin, cyfluthrin, and lambda-cyhalothrin) have been introduced to control programs of bed bugs since 2000 and in the past decade of new millennium. In virtue of the enhanced contamination and resurgence of bed bugs, the application of these insecticides, along with OPs, Cs, phenylpyrazoles (e.g. fipronil), and neonicotinoids (imidacloprid), was escalated.

Studies on the Middle East are scarce and limited to a few reports of the common bed bug from Iran [32–34], Israel [24, 35], and Kuwait [36], and of the tropical bed bug from Israel [6] and Iraq [7]. Aside from these reports, there is documentation of the insensitivity of *C. lectularius* to malathion, diazinon, and lambda-cyhalothrin in the northeast of Iran [37] and also V419L, L925I, and I936F *kdr-*type mutations in different populations of the common bed bug from Israel [24]. Despite numerous reports of *C. lectularius* resistance to pyrethroids, there is scant information on the resistance status of the tropical bed bug in the Middle East. Owing to recently witnessed emerging character, socioeconomic impact, and potential health hazards of this pest, understanding the role of insecticide resistance in the occurrence of this species is necessary for its monitoring and management. Moreover, this information is essential to diminish and eliminate the re-infestation of this pest that accompanies its presence. Therefore, the aims of the present study were to determine the permethrin resistance status and to elucidate the role of target site insensitivity in pyrethroid resistance in various populations of the tropical bed bug. To achieve this goal, the bioassay tests and molecular surveys were conducted to assess the pyrethroid resistance, characterize the pyrethroid receptor sites in *vgsc* gene, and detect the *kdr*-associated mutations and their distribution in the populations of the tropical bed bug in Iran.

## Methods

### Bed bug sampling

**Fig. 1:**
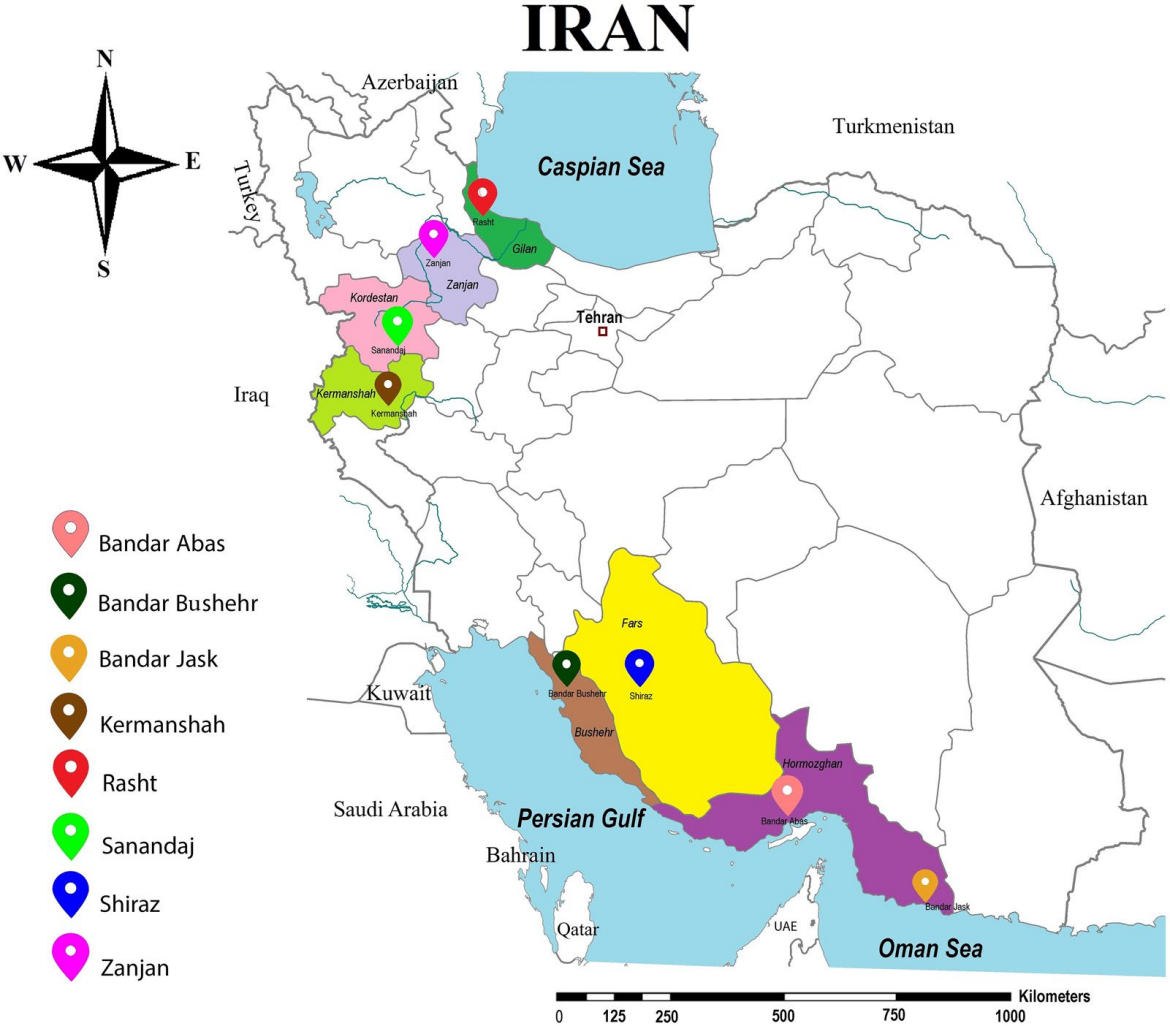
Collection sites of tropical bed bug specimens in Iran

Live bed bugs were collected from residential areas, including villas, complexes, apartments, hotels, motels, inns, and dormitories, in various parts of Iran (Fig. 1), from 2015 to 2020. In this decade, different insecticides, pirimiphos-methyl, fenitrothion, propoxur, bendiocarb, permethrin, deltamethrin and lambda-cyhalothrin, have been used to control bed bugs in study areas. Morphological identification of the adult tropical bed bug was carried out under microscopic observations, focusing on the pronotum, paragenital sinus, hind tibial pad, and parameter characters [38, 39].

### Bioassay test

Adult bed bugs from the selected sites were subjected to insecticide bioassay adapted to previously applied methods [19, 22, 23, 25, 28, 31, 36, 40–45]. The permethrin 0.75%, dosage used in previous study [42], was considered suitable for resistant-population discrimination, due to the lack of diagnostic dose for tropical bed bug and insufficiency of the permethrin 0.25% (*C. lectularius* recommended diagnostic dose) in detection of the mortality and knockdown values [26]. Bed bug samples were exposed to permethrin (0.75%)-impregnated filter papers, supplied by USM/01 (www.inreskit.usm.my). At least three groups of 10–15 mixed-sex bed bugs from each selected site were put in contact with impregnated filter papers disks (8-cm diameter), tightly fitted in sterile 8-cm Petri dishes, and kept in dark conditions in 40% relative humidity at 24 ± 2 °C for 48 h. Three groups of 10 bugs were also exposed to silicone oil-impregnated papers and were used as negative controls. In the studied groups, the knockdown and mortality data were recorded at 24 and 48 h, and in the selected populations, the knockdown samples were scored 2–4-hourly for 24 and 48 h. At the end of tests, the dead, knockdown, and alive samples were stored at −20 °C for further investigation. The death of bed bugs was determined by the absence of leg and antennae movements when the specimen was gently inspected with forceps. Abbott’s formula was applied if the negative control mortalities were between 5 and 20%. Permethrin resistance status was evaluated by using the classification determined by the WHO, in which > 98% mortality indicated susceptible, and less than 98% suggested the presence of resistance [46]. The knockdown time 50 (KT_50_, the time by which 50% of bed bugs were paralyzed) and KT_90_ (the time by which 90% of bed bugs were paralyzed) values were estimated from knockdown data by probit analysis using PriProbit version 1.63 software [47]. Knockdown time ratios (KR_50_ and KR_90_) were calculated by comparing the rates of KT_50_ and KT_90_ in the studied populations of tropical bed bugs with those of the *C. lectularius* Kh1 strain.

### Genomic DNA (gDNA) extraction

The gDNA of the samples was extracted as described before [48, 49]. In brief, the samples were stored at −70 °C for 1 h. The frozen bed bugs were then placed individually in 1.5-ml tubes and homogenized in 400 µl of a lysing buffer (100 mM of Tris HCl [pH 8.0], 0.5 mM of NaCl, 10 mM of EDTA, and 1% W/V SDS) with glass beads by the aid of an electric homogenizer. Subsequently, 20 µg of proteinase K was added to the homogenates before incubation at 55 °C for 3 h. Next, 100 µl of 8 M potassium acetate was added to each tube, mixed gently, and kept on ice bath for 10 min. The samples were spun at 4000×*g* for 10 min, and the supernatants were transferred to fresh tubes. One milliliter of ice-cold absolute ethanol was added to each tube, followed by spinning at 8000×*g* for 10 min. Pellets were washed in 0.5 ml of 70% ethanol, before an eventual spinning at 8000×*g* for 10 min. The final pellet was dried at room temperature and re-suspended in 50 µl of TE buffer.

### PCR assay

Fragment amplification was carried out using the primers FKDR1 (GTCCGTGGCACATGTTGTTCTTCA) and RKDR1 (CTGATGGAGATTTTGCCACTGATGC) for the first region (the domain IS6 and the partial domain I–II linker) and FKDR2 (GGAATTGAA GCTGCCATGAAGTTG) and RKDR2 (TGCCTATTCTGTTCGAAAGCCTCAG) for the second region (the domain II and the partial domain II–III linker). These primers were designed from the nucleotide sequences of PCR products that their primers have been proposed in previous studies [14, 23, 25, 44]. The PCR reaction contained 12.5 μl of Taq DNA Polymerase 2X Master Mix RED (Ampliqon, Denmark), 1 μl of both forward and reverse primers (10 pM), and 2 μl of DNA template in a total volume of 25 μl. PCR for both reactions were performed under the following conditions: 95 °C for 2 min, following by 40 cycles of 94 °C for 20 s, 58 °C for 3 s, and 72 °C for 2 min, and the final extension of 70 °C for 10 min.

### DNA sequencing and analysis of PCR products

To monitor the putative *kdr* mutations in the *vgsc* gene, some of the amplified products were randomly selected. The products were purified by PCR Clean-Up Kit (SinaClon, Iran) and sequenced bi-directionally by Macrogen (Seoul, Korea) using the forward and reverse specific primers. The nucleotide sequences of samples were aligned by Clustal Omega (https://www.ebi.ac.uk/Tools/msa/clustalo/) and analyzed using BioEdit [50] and MEGA X [51] software. The *kdr* mutations were confirmed by comparing the amino acid sequences of samples with reference *C. lectularius* alignments (GenBank Accession Nos. FJ1997, GU123927, and GU123928). The nucleotide sequences in some of the samples were submitted to the NCBI GenBank.

### SYBR Green qRT-PCR assay

Two singleplex real-time PCR assays were developed to genotype the pyrethroid resistance in the second region of the *vgsc* gene. The first assay targeted the mutation in the domain IIS4-5, and the primers were designed to flank the following nucleotide substitution in 918 locus. Reaction with the forward (FKDR2) and reverse (KDR; 5’CTCCATCAGGGAATCTATCCATGCT) primers yielded a 467-bp amplicon. The second assay targeted the domain IIS6 mutation, L1014F, and primers used in the reaction were KDF (AGCATGGATAGATTCCCTGATGGAG) and RKDR2, which produced a 370-bp amplicon. Each reaction (20 μl) contained 4 μl of sample gDNA, 900 nM of each primer, and 10 μl of Ampliqon SYBR Green Master mix High Rox (Ampliqon, Denmark). The sequenced amplicons of *vgsc* gene of the mutant tropical bed bug (with M918I and L1014F mutations), Kh1 strain of the common bed bug (without deduced double mutations), and ddH_2_0 were used as positive control of the resistant alleles, positive control of the susceptible alleles, and negative control, respectively. The reaction was started with 95 °C hold for 10 min, followed by 40 cycles at 95 °C for 15 s, 60 °C for 20 s, and 72 °C for 25 s and last hold step for 30 s at 90 °C. Melt curve analysis initiated from 60 to 90 °C at a rate of 0.1 °C /S, and the change in fluorescence of SYBR Green was measured continuously on the green channel of the ABI StepOnePlus RT-PCR system (Applied Biosystems, USA). Melt curves were generated in the StepOne 2.3 software (www.lifetechnologies.com). Gain optimization of all tubes was carried out before the melt analysis to obtain high-quality melting peaks. Each specimen was named as resistant (RR), susceptible (SS), and heterozygous (RS) according to the melt curves.

### TaqMan qRT-PCR assay

The TaqMan PCR assays were conducted to detect the M918I mutation in bed bug samples. For these assays, the forward (CHPF; GCTCAGGGTGTTTAAGCTGG) and reverse (CHPR; CTGCATTCCCATCACAGCGA) primers, together with the wild-type CHSP (HEX-labeled probe; CCACTGTTCTGCCCATAATG) and the mutant-type CHRP (FAM-labeled probe; CCACTGTTCTGCCTATAATG), were designed from the sequences of specific PCR products of the second region using Primer Express version 2.0 software (Life Technologies, USA). The 5ʹ HEX- and FAM-labeled probes were also comprised of a non-fluorescent black hole quencher (BHQ) binder at their 3ʹ ends. PCR assay reactions (25 μl) contained 3 μl of gDNA, 12.5 μl of Probe-High Rox qPCR Master Mix (SinaClon, Iran), 700 nM of each primer, and 250 nM of each probe. Reactions were run on an ABI StepOnePlus RT-PCR system using temperature cycling conditions of 10 min at 95 °C, followed by 40 cycles of 95 °C for 10 s and 60 °C for 45 s. Increase in FAM and HEX fluorescence was monitored in real-time by acquiring each cycle on the blue (494 nm excitation and 518 nm emission) and green (535 nm excitation and 556 nm emission) channels, respectively, to aid in assigning genotypes.

## Results

A total of 1377 adult bed bugs were collected from the study districts (Fig. 1), and all were identified as *C. hemipterus*. From the collected samples, 437 individuals were selected randomly and subjected to bioassay survey. Study populations were found to be resistant to permethrin when tested in contact bioassay within the 48-h exposure to the mentioned insecticide. The cumulative mortality rates in these populations were in the range of 30.7–38.7% and 58.4–77.4% in 24 and 48 h, respectively (Table 1). Among *C. lectularius* Kh1 strain, the corrected mortality rate within 24 h was obtained at > 99.0%. Results suggested that all the populations of *C. hemipterus* were fully resistant to permethrin. The knockdown rates of the tropical bed bug populations ranged from 32.2 to 46.6% and from 61 to 83.8% in the first and second days, respectively. There was no significant difference in the knockdown and mortality rates between studied populations (*χ*^2^ < 0.41, *df* = 6, *p* = 0.99). The low response to permethrin also occurred in minimum knockdown times. The KT_50_ and KT_90_ values in Zanjan and Rasht populations of the tropical bed bug varied in the range of 27.9–29.0 and 82.8–104.42 h, respectively, while these values were detected as 5.39 and 135.55 h in the common bed bug strain. The KT values obtained for *C. lectularius* population did not follow normal distribution for knockdown to log time (*χ*^2^ > 24.40, *df* = 8, *p* < 0.001). The KR_50_ and KR_90_ values among the studied populations ranged from 5.17 to 6.17 and from 5.35 to 7.58, respectively (Table 2). The results indicated *kdr* development among the studied populations.

**Table 1:** The percentage of knockdown and mortality in populations of the tropical bed bug exposing to permethrin 0.75% in 24 and 48 h

District	Geographic coordinate		*N*	Knockdown rate (%)		Mortality rate (%)	
	Latitude	Longitude		24 h	48 h	24 h	48 h
Bandar Abbas	27.1556–27.2024	56.2461–56.2510	65	32.2	61.5	30.7	58.4
Bandar Bushehr	29.9004–29.9183	50.8533–50.9308	46	36.8	63.0	32.6	56.2
Bandar Jask	25.6594–25.6603	57.7872–57.7904	42	38.0	76.2	35.7	69.0
Kermanshah	34.3113–34.3259	47.0608–47.0900	31	41.9	83.8	38.7	77.4
Rasht	37.2708–37.2747	49.5839–49.5912	37	40.5	75.6	35.1	70.2
Shiraz	29.6165–29.6307	52.4578–52.6189	56	42.8	80.4	35.7	75.0
Zanjan	36.6635–36.6867	48.4522–48.5316	73	46.6	72.6	36.9	68.5

**Table 2:** Regression parameters and expected minimum knockdown time (hours) in selected strains of tropical bed bug (*C. h*) and common bed bug (*C. l*) exposing to permethrin 0.75%

Bed bug stain	*N*	*a*	*b*	*X*^2^ (*df*)	H	KT_50_ (95% CL) hrs	KT_90_ (95% CL) hrs	KR_50_	KR_90_
*C. h* Ra1	37	− 8.95	2.77	3.16 (11)	0.25	28.54 (25.61–32.09)	82.81 (66.19–115.45)	5.29	5.35
*C. h* Zn1	36	− 8.04	2.48	0.90 (11)	0.08	29.03 (25.73–33.18)	95.36 (73.28–142.42)	5.38	6.13
*C. h* Zn2	39	− 7.21	2.23	1.18 (11)	0.10	27.9 (24.53–32.22)	104.40 (78.24–162.2)	5.17	6.71
*C. h* Zn3	36	− 7.72	2.33	1.01 (11)	0.09	33.25 (32.49–39.07)	117.99 (85.48–195.81)	6.17	7.58
*C. l* Kh1	33	− 7.09	2.81	24.41 (8)	3.05	5.39 (3.73–7.27)	15.53 (11.79–238.96)	–	–

*N* = number of individuals, *a* = intercept of the regression line, *b* = slope of the regression line, KT = minimum knockdown time, *df* = degrees of freedom, *H* = heterogeneity on the Chi-square goodness of fit test, KT_50_ = median knockdown time in hours, CL = confidence limits, KR = Knockdown time ratio, Ra = Rasht, Zn = Zanjan, Kh = Khodabandeh

**Fig. 2:**
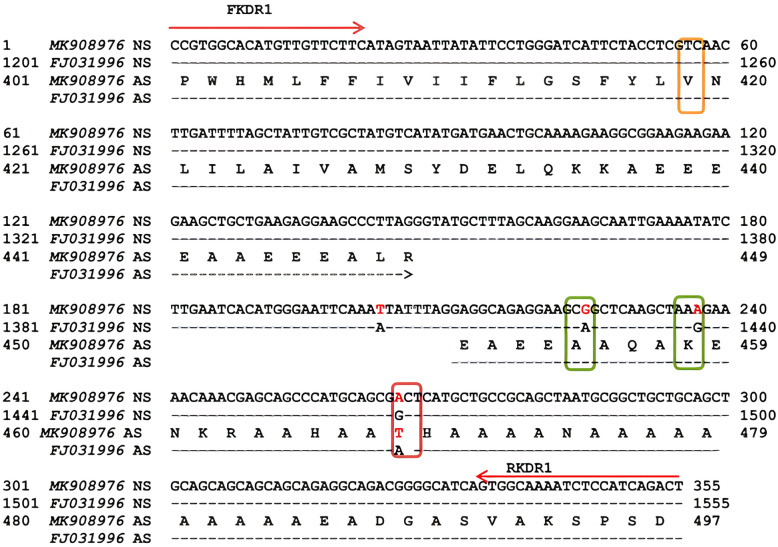
Alignment of the domain IS6 and partial domain I–II linker fragment of the *vgsc* gene of *C. hemipterus* with Accession No. MK908976 against the reference *vgsc* gene of *C. lectularius* wild type with Accession No. FJ031996. The mutation sites are indicated in vertical boxes; orange, no point mutation at V419 site, green, silent mutation with transition at A454 and K458 sites, and red, missense mutation with transition at A468T site. Nucleotides 1–147 include exon I, 148–211 intron I, and 212–355 exon II. NS and AS represent nucleotide sequence and deduced amino acid sequences, respectively. The location of primers are indicated with red arrows

**Fig. 3:**
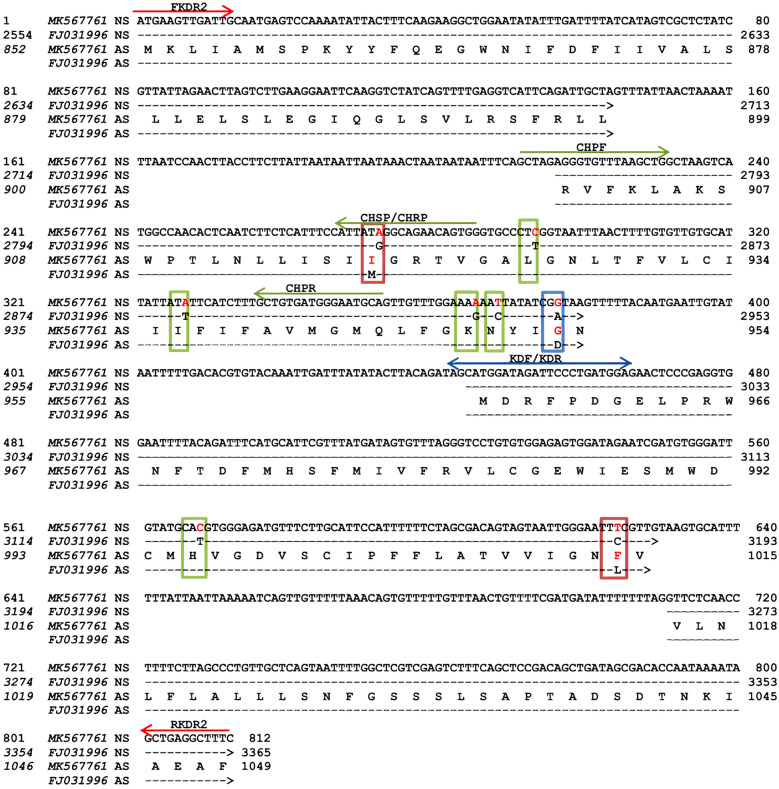
Alignment of the domains IIS1–S6 and partial domain II–III linker fragment of the *vgsc* gene of *C. hemipterus* with Accession No. MK567761 against the reference *vgsc* gene of *C. lectularius* wild type with Accession No. FJ031996. The mutation sites are indicated in vertical boxes; red, nonsense mutation at M918 and L1014 sites, green, silent mutations with transition (at L925, K949, N950, and H995 sites), and transversion (at L936 site), and blue, novel non-synonymous mutation with transition at D953 site. Nucleotides 1–144 include exon I, 145–217 intron I, 218–380 exon II, 381–445 intron II, 446–628 exon III, 629–711 intron II, and 712–812 exon IV. NS and AS represent nucleotide sequence and deduced amino acid sequences, respectively. The location of primers are indicated with red (conventional PCR), blue (SYBR Green real-time PCR), and green (TaqMan real-time PCR**)** arrows

Two regions of *vgsc* gene, containing key mutations of *kdr*-type resistance to PYs, were investigated in 313 samples of bed bugs. Fragments of the domain IS6 and the partial domain I–II linker of *vgsc* gene were amplified in 78 samples. A total of 14 amplicons (two samples from each population) were randomly selected for sequencing. The amplified fragment was 355 bp in size and contained an intron (64 bp) and two 3ʹ (147 bp) and 5ʹ (144 bp) exons encoding 97 amino acids (Fig. 2). The second region of the *vgsc* gene, the domain II and the partial domain II–III linker, was also amplified in 225 individuals, and 88 samples (11 amplicons from each population) were selected randomly and subjected to sequencing. Analysis of the sequences showed an 812-bp fragment containing three introns, I (73 bp), II (65 bp), and III (83 bp), and four exons, 3ʹ (144 bp), internal I (163 bp), internal II (183 bp), and 5ʹ (101 bp), and encoded 198 amino acids (Fig. 3). In samples subjected to DNA sequencing, no polymorphic site was found in the nucleotides alignment of the PCR products in both pyrethroid receptor sites. The consensus sequences were blasted in the NCBI and compared with the GenBank database. Analysis of these sequences against the reference strain of common bed bug, showed 98% identity in exon section, differing in three nucleotides within the first region (Fig. 2). Similarly, the exon nucleotide sequences of the second region of *C. hemipterus* reflected 95% identity with *C. lectularius*, with eight-nucleotide difference (Fig. 3). This analysis also showed that the fragments of the second region in *C. lectularius* Kh1 strain were complete similar with domain IIS1–S6 of reference common bed bug (Accession Number FJ031996). Nucleotide sequences of the studied fragments were submitted and are accessible in the NCBI GenBank with the Accession Numbers MK567761–MK567778, MK908866, MK908869–MK908921, and MK908967–MK908976.

To identify mutation within the studied populations, the nucleotide sequences of the exon at the first region were translated into protein and compared with reference sequence. No amino acid change was evident at V419 site in *C. hemipterus*, but A468T substitution was found in this region (Fig. 2). This replacement (A↔G transition) exhibited on ACT to GCT substitution at the first base of the A468 allele was located in the internal linker of the domain I–II, which caused amino acid change from *alanine* to *threonine* (Ala to Thr). Before the A468T substitution, two silent mutations with a transition at A454 and K458 sites occurred in the first region. Despite little *kdr* mutation evidence in the first region of the deduced gene, several mutations were identified in the second region. In this region, three transitions with change in amino acid at sites I918 (*isoleucine*/I: ATA), G953 (*glycine*/G: GGT), and F1014 (*phenylalanine*/F: TTC) were presented in *C. hemipterus*, compared to sites M918 (*methionine*/M: ATG), D953 (*aspartic acid*/D: GAT), and L1014 (*leucine*/L: CTG) in *C. lectularius* reference gene. The remaining mutations in the second region of *C. hemipterus* were silent. These mutations included transition at L925, K949, N950, and H995 sites and transversion at L854 and I936 sites (Fig. 3).

**Fig. 4:**
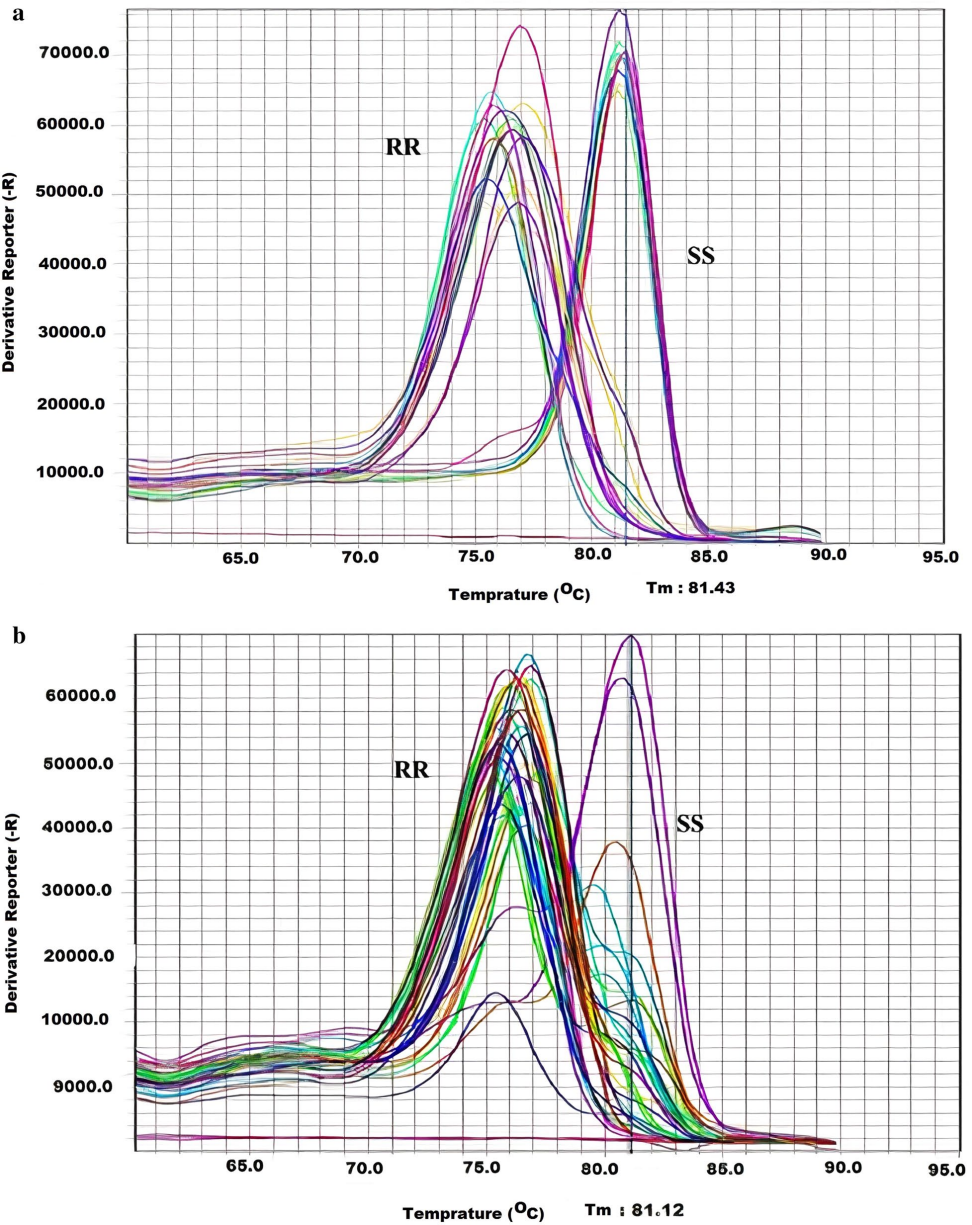
Melting curve of the *vgsc* gene fragments obtained with SYBR Green real-time PCR assay in studied samples of bed bugs. **a** Fragments with position 918, **b** Fragments with position 1014, (RR) mutant type of *C. hemipterus*, (SS) *C. lectularius* Kh1 strain without mutation in 918 and 1014 loci and similar with the domain IIS1–S6 of common bed bug (Accession No FJ031996). The violet curves and baselines represent the positive control (reference sequences of *C. hemipterus* and *C. lectularius*) and negative control, respectively. The curves on the right side of the plots, marked with SS, are common bed bug specimens and the curves on the left side of the plots, marked with RR, are samples of tropical bed bugs

Melting peaks obtained from SYBR Green qRT-PCR assay were analyzed in 70 and 25 specimens of *C. hemipterus* and *C. lectularius*, respectively. All the tropical bed bugs showed a single peak at 76.5 °C and 76 °C in the domain IIS4–5 and IIS6 fragments, respectively. Although a single peak was also presented in all of the common bed bug samples for these fragments, the melting temperature in this bed bug was more than *C. hemipterus* and reached 81.5 and 81.0 in the deduced fragments, respectively. The I918/I918 and F1014/F1014 resistant homozygous alleles were visible in all the *C. hemipterus* samples. However, the M918/M918 and L1014/L1014 susceptible homozygous alleles were presented in all the common bed bug individuals (Fig. 4).

**Fig. 5:**
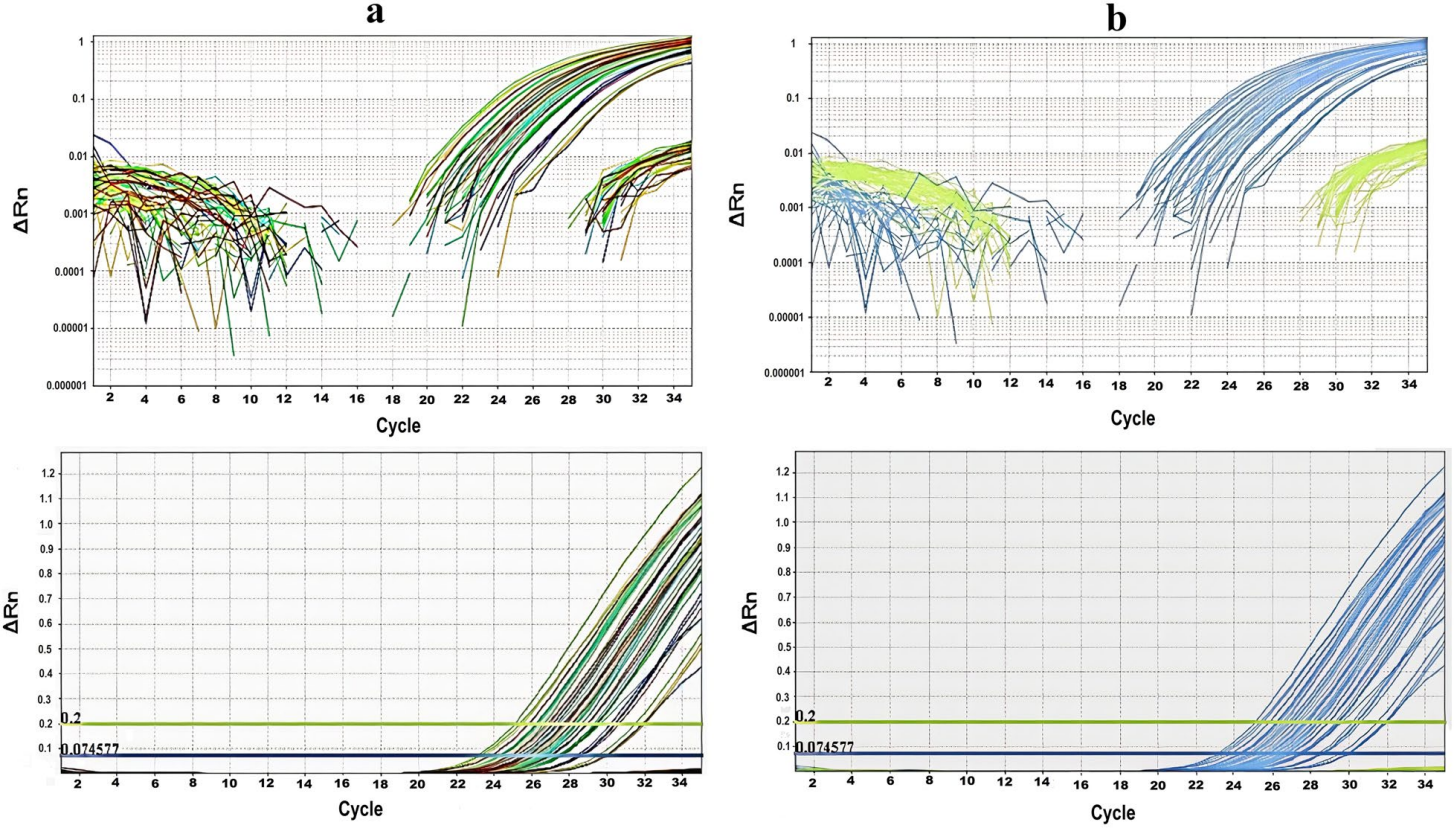
Amplification plots of the *vgsc* gene fragment of studied tropical bed bugs using TaqMan real-time PCR. **a** Amplification of the mutant (with M918I mutation) and wild-type DNA fragments in both samples. **b** Amplification of mutant and wild-type fragments in all samples. The solid blue and green lines marked with 0.074577 and 0.2 represent the threshold cycle (Ct) in the mutant and wild groups, respectively. The ΔRn in the *Y* axis is the difference of fluorescence between the sample and baseline. The baseline of mutant and wild groups (represented as blue and green lines, respectively) is the line at which there are no background fluorescence peaks crossing over it

TaqMan real-time PCR assay was performed for 840 samples of the studied populations of the tropical bed bug. The M918I mutation was presented in all the samples, and all of the studied populations carried resistant *kdr* alleles (Fig. 5).

## Discussion

This is the first study that widely surveyed the field strains of tropical bed bug in Iran via bioassay and molecular analysis. There has previously been no available information regarding in *kdr* and sequence data of *vgsc* gene of this species in studied areas. Therefore, generating relevant bioassay and genetic data was of high priority for investigations focusing on bed bug control in this country.

Because we did not have a standard susceptible strain, as a limitation of this study, comparison of the bioassay data between studied populations revealed low levels of knockdown and mortality rates to permethrin in all of the field strains of tropical bed bug. Even though the knockdown and the mortality rates are not indicators of the same mechanisms of resistance and evolutionary selection, the correlation between these indexes is strong enough to identify permethrin resistance in the field populations. These values cannot be confidently related to the permethrin resistance phenotype and to identify the actual values further research is suggested with the provision of sensitive strain.

Similar phenomenon in this study, i.e. resistance to insecticides, was found in the field strains of *C. hemipterus* to different pyrethroids (bifenthrin, cypermethrin, esfenvalerate, and etofenprox) from Thailand [42], DDT and deltamethrin residues from Malaysia [45], a mixture of deltamethrin and imidacloprid from Australia [52], and permethrin and deltamethrin from Sri Lanka [26]. These conditions were also represented in different populations of the common bed bug for pyrethroids from USA [23], permethrin and deltamethrin from Australia [53], allethrin from Sydney [44], imidacloprid and β-cyfluthrin from Malaysia [54, 55], several neonicotinoids (imidacloprid, acetamiprid, thiamethoxam, and dinotefuran) from USA [56], bifenthrin from Germany [57], a mixture of deltamethrin and imidacloprid from Australia [53], and bendiocarb from Paris [58]. Based on the bioassay results and the findings of previous studies [26, 42, 45, 52], all the bed bug populations in the present study manifested resistance to permethrin. Thus, the reconsideration of control methodologies is of paramount importance in the studied areas.

From the bioassay data, it was determined that *C. hemipterus* had higher resistance to permethrin compared to the common bed bug, and KR values were found to be 5–7 in this bed bug. Due to the lack of a susceptible strain, the main limitation of this study, the estimated values of KR using *C. lectularius* Kh1 field samples, cannot be a real value of resistance factor, as they are overestimated. Despite the absence of M918I and L1014F mutations in common bed bug samples, the possibility of V419L mutation [9, 19, 22–24], detoxification of enzymes [9], and heterogeneity of field samples could enhance the baseline susceptible data in this bed bug. Nevertheless, the higher value of KR, explaining the high frequency of *kdr* alleles, is strong evidence for the development of pyrethroid resistance. Therefore, the inclusion of resistance management in modified control programs is an important issue that needs to be taken into account.

In the present study, quantitative real-time TaqMan and SYBR Green PCR assays confirmed *kdr*-related mutations in the populations of tropical bed bug. There are numerous applications of these techniques in gene expression, DNA quantification, and genotyping, as well as in detecting and monitoring insecticide resistance in vector populations. Simultaneous application and visualization of newly formed DNA amplicon by these methods facilitate fast, high-throughput detection and quantification of target DNA sequences in different matrices. In addition, there is no need for further manipulations, such as electrophoresis and densitometry, which render these methods be safe in terms of avoiding contaminations. Other advantages of qRT-PCR methods include a wide dynamic range for quantification and multiplexing of amplifications of several targets into a single reaction. Despite the lack of susceptible strain of tropical bed bug, the similarity of nucleotide sequence of common bed bug Kh1 strain at 918 and 1014 loci with the sensitive samples of tropical bed bug, SYBR Green assay is suggested to identify the frequency of mutant type at 918 locus, and both of wild and mutant genotypes at 1014 site in future studies. However, the presence of nucleotide polymorphisms in the downstream of the 918 locus may cause challenges in the analysis of the wild genotype.

With the help of the SYBR Green and TaqMan real-time PCR assays, the major *kdr/super-kdr* mutations were identified in all of the tropical bed bug samples. The role of L1014F mutation in *kdr-*type resistance, reduction of target site sensitivity to pyrethroids, and M918I mutation with *super-kdr* resistance in *C. hemipterus* and several other insects have been affirmed in numerous earlier studies [9–13, 15, 16, 21, 25, 59–62]. From the result of this study and those of previous investigations, it can be concluded that the populations of tropical bed bug in the studied areas could have a high level of resistance to pyrethroids. This situation calls for a decrease in reliance on pyrethroids and highlights the need for alternative insecticides with different modes of action or alternative approaches to make bed bug control more effective.

DNA sequencing identified the double mutation, M918I, and L1014F, in all of studied populations. Because *kdr* and *super-kdr* genotypes are presented together, a very high resistance is expected in the studied areas. Although the traditional bioassay in the present study showed resistance to permethrin, in order to identify the actual level of resistance, it is necessary to conduct further studies using a standard bioassay test. The occurrence of M918I and L1014F mutations signifies the emergence and expansion of strong pyrethroid resistance of tropical bed bug in different areas of Iran. Two hypotheses could explain the emergence and expansion of this pest. In the first hypothesis, the homogenous strains with *super-kdr* alleles, historically undergo strong PYs selection pressure, might have been introduced to Iran in the early third millennium and subsequently expanded rapidly throughout the country due to human-mediated movement. The second hypothesis for the expansion of strongly resistant strains includes the adaptive response to the selective pressure of applied PYs and their inability to control this emergent pest. Previously, various parts of the study areas were covered by OCs in the 1960s, with the implementation of the malaria control program. After controlling malaria in the northern half of Iran, the southern parts were sprayed with OPs and Cs pesticides until the 1990s. A number of villages in the northern parts of the country were subjected to PYs, Cs, and OPs insecticide pressure in the 2010s, due to the implementation of parasite control plan by the Iranian Veterinary Organization, Ministry of Agriculture Jihad. Moreover, contaminated rooms were sprayed with Cs, OPs, and PYs insecticides by private pest control agents over the past three decades. In the studied populations, target site insensitivity and *kdr*-mediated resistance mechanism may be subjected to selection, as DDT was introduced for the first time. Since DDT and PYs have a similar mode of action, cross-resistance between them, the selection pressure has continued with the application of pyrethroids for controlling public health pests and pyrethroid-treated bed nets. However, after the control of malaria and the cessation of vector control programs, the selective pressure decreased during the last three decades. For the reason that there is not a huge selection pressure to justify the emergence of such homogeneity in the *kdr* and *super-kdr* allele, the probability of implying the adaptive response to selective pressure hypothesis is lower, and it is more likely that the homogenous strains with *super-kdr* allele were introduced to Iran. Although the double mutation (M918I and L1014F) *kdr* allele was found in populations from Australia, India, Thailand, and China [19, 25, 59], the origin of this invasive pest in Iran is under question because the corresponding gDNA sequences of the target gene are not publicly available. Additional complimentary studies with microsatellite or mitochondrial markers, such as the study of Zhao et al. [59], are suggested to clarify this hypothesis via investigating more samples from different populations. Our study was not designed to seek the point of the origin or introduction of pyrethroid resistance into bed bug populations. Nevertheless, this resistance may have a multifunctional origin, and mechanisms such as metabolic breakdown and efflux may implicate this issue [9]. Further epigenetic studies with more population and additional molecular markers are needed to come to a final conclusion on the origin of bed bug resistance in the study areas. Moreover, the presence of *kdr* resistance and the inability of PYs to control this emergent pest necessitate reconsidering control methodologies and applying products against this pest. Indeed, the application of the integrated pest management measures and determination of the ecological, social and biological risk factors, which play a key role in bed bug infestation, are important issues that need to be considered in developing control programs against this invasive pest.

Multiple alignment of DNA sequence from *C. hemipterus* with that of *C. lectularius* presented the A468T and D953G mutations, in addition to the M918I and L1014F mutations, in studied populations. The 468 locus in the intracellular linker of the domain IS6–IIS1 is the hypervariable site in different species of insect. Therefore, the nucleotide variation in this locus may reflect the difference between two bed bug species. On the other hand, known *kdr* mutations in this region are rare, and only E435K at the beginning and C785R mutations at the end of the I–II linker were identified in pyrethroid-resistant strains of the German cockroach, *Blattella germanica* [18]. Accordingly, the A468T substitution might be related to insecticide resistance in the tropical bed bug. The D953G mutation, located in the linker of the domain IIS5–S6, has been reported from pyrethroid-resistant strains of *C. hemipterus* in Thailand [25]. This mutation, which is equivalent to L936F mutation in corn earworm, *Helicoverpa zea*, has been verified to reduce the sensitivity to pyrethroids [18, 63]. The *vgsc* gene regions, where A468 and D953 are located, are major sites for the influence of PYs, and several point mutations clustering in these regions affect resistance to pyrethroids [9–11, 13, 15, 20, 21]. Therefore, this study highlights that the D953G and A468T substitutions could have additive effects on main *kdr* mutations in pyrethroid resistance, though other forms of resistance (cuticular and detoxification) cannot be excluded. Ultimately, the function of the A468T and D953G variations needs to be tested by the construction of mutant transcript in further studies, to confirm that this construction is actually a *kdr* mutation against the pyrethroid resistance.

Unlike the pyrethroid-resistant strains of *C. lectularius* in the present study, the L925I, I936F, V419L, and other putative *kdr* mutations in the IS6 and the partial domain I–II linker were not found in *C. hemipterus*. The existence of M918I and L1014F mutations in the tropical bed bug expresses the specific responses of this species to the selective pressure of PYs. These adaptive responses may be related to the innate immune system and DNA damage repair pathways in *C. hemipterus* [64, 65].

The aim of this investigation was to determine the *kdr* mutation in different populations of tropical bed bug, and, therefore, several point mutations, including A468T, D953G, M918I, and L1014F, were identified. Similar to the finding of our study, previous investigations have documented the D953G, M918I, and L1014F mutations in other populations of bed bugs and other insects [9, 13, 15, 25, 61, 62]. However, owing to the mechanisms of post-transcriptional regulatory variation in organisms, mRNA editing is possible [66]. Perhaps, the RNA sequence can be altered from the encoding DNA template, which can potentially lead to a change in the actual DNA expression of the *kdr* mutation. There are known surveys showing that insects use RNA editing to create variations in the *vgsc* gene [67, 68]. Consequently, future studies based on both DNA and cDNA sequencing would strengthen the findings of this study.

The results of the current study showed the high frequency of *kdr* mutations and pyrethroid resistance in different populations of tropical bed bug in the studied areas. Developing resistance management plans and applying effective tactics [2, 69] are essential to lessen the bed bug infestation and frequency of mutant alleles. Accordingly, the use of insecticides with different modes of action and intensive application of pyrethroids with insect growth regulators (IGRs) are recommended to control the tropical bed bug. Moreover, integration of non-chemical methods [70], i.e. applying multidisciplinary control methods to prevent pest entry, limiting access to feed on hosts and shelters, vacuuming, using impregnated covers and mattresses, and employing heat in form of dry steam and cold, are suggested for the management of this invasive pest.

Given that the passive host/furniture-mediated dispersal is the common *modus operandi*, and the active dispersal is restricted to within buildings, in addition to the mentioned control tactics, other strategies should also be considered in developing control plans, such as educating travelers to recognize bed bugs and avoid carrying them from place to place in infested luggage, training residents to identify pest and take necessary actions when infestation is suspended, providing guidelines for accommodation suppliers in how to make rooms less bed bug friendly, and establishing instructions and rules for importing and trading of second-hand goods, furniture, and clothing [2, 63, 71]. Altogether, in setting up management plans for bed bugs, the biological and socioeconomic risk factors, as well as the side effects of current control measures against public health pests, should be taken into account.

## Conclusions

The distribution map of tropical bed bug has been expanded in subtropical and temperate areas. The high sensitive/specific, rapid, and cost-effective qRT-PCR assays developed in the present study are suggested for detecting the allele frequencies of *kdr*-type mutations in different populations of tropical bed bug. The lack of mortality and knockdown effects in exposure to permethrin, together with the presence of *kdr*-type mutations in *vgsc* gene, indicates the expansion of *super-kdr*-resistant populations of tropical bed bug in various areas of Iran. Further complimentary studies with microsatellite or mitochondrial markers are proposed to explain the evolutionary origin of this resistance. In addition, reconsideration of control strategies and product registration against this resurgent pest is advised for managing resistance and decrease of resistant alleles. Obviously, application of effective insecticides, as well as the consideration of biological and socioeconomic risk factors and the side effects of current public pest control measures, need to be taken into account in the establishment of revised control strategies.

## Data Availability

All data generated or analyzed during this study are included in this published article. The newly generated sequences were submitted to the GenBank database under the Accession Numbers MK567761–MK567778, MK908866, MK908869–MK908921, and MK908967–MK908976.

## Abbreviations

Cs: Carbamates
gDNA: Genomic DNA
*kdr*: Knockdown resistance
KR: Knockdown resistance ratio
KT: Knockdown time
OCs: Organochlorine compounds
Ops: Organophosphates
NCBI: National Center for Biotechnology Information
PYs: Pyrethroids
qRT-PCR: Quantitative real-time polymerase chain reaction
*vgsc*: Voltage-gated sodium channel gene
VGSC: Voltage-gated sodium channel protein
